# Autoimmune thyroiditis in antinuclear antibody positive children without rheumatologic disease

**DOI:** 10.1186/1546-0096-8-15

**Published:** 2010-05-05

**Authors:** Kathryn S Torok, Thaschawee Arkachaisri

**Affiliations:** 1 University of Pittsburgh, Children's Hospital of Pittsburgh, Department of Pediatrics, Division of Rheumatology, Pittsburgh, Pennsylvania, USA; 2 Previous address: University of Pittsburgh, Pittsburgh, PA 15224, USA; 3 Current address: Rheumatology and Immunology Services of the Department of Paediatric Subspecialties at the KK Women's and Children's Hospital, Singapore

## Abstract

**Background:**

Children are commonly referred to a pediatric rheumatology center for the laboratory finding of an Anti-nuclear antibody (ANA) of undetermined significance. Previous studies regarding adult rheumatology patients have supported an association between ANA and anti-thyroid antibodies, with the prevalence of thyroid antibodies being significantly higher in patients referred to a rheumatology center for an ANA without evidence of connective tissue disease compared to the general population. The purpose of the present study was to determine the frequency of thyroid antibodies in children referred to a pediatric rheumatology center for a positive ANA without evidence of a connective tissue disease.

**Methods:**

A retrospective chart review was performed on children who were referred to our pediatric rheumatology center between August 2003 and March 2007 for positive ANA with concurrent thyroid antibody and thyroid function tests performed who did not fulfill criteria for a specific connective tissue disease. Laboratory and clinical features were recorded and analyzed. Mean and standard deviation were used to describe continuous data. Chi-square or Fisher's exact tests were used to compare proportions between variables.

**Results:**

One-hundred and four ANA-positive patients with concurrent thyroid studies were evaluated (88% female, 93% Caucasian, mean age 11.9 ± 4.0 years). Half of patients had an ANA titer ≥ 1:320. The ANA pattern was speckled in 60% of the patients. Thyroid antibodies were detected in 30% of the patients. Anti-Thyroglobulin (ATG) was detected in 29% and Anti-thyroid peroxidase (ATPO) in 21% of the patients; of these children, 14% had hypothyroidism. ANA pattern and titer were not associated with anti-thyroid antibody positivity.

**Conclusion:**

Thyroid antibodies associated with chronic lymphocytic thyroiditis, ATG and ATPO, were detected significantly higher in ANA-positive children without a rheumatologic condition (30%) as compared to the general pediatric population (1.3 - 3.4%). ANA titer and pattern did not help predict the presence or absence of thyroid antibodies. Given the high frequency of thyroid antibodies and increased risk of developing hypothyroidism over time, routine evaluation of ATG and ATPO with thyroid function tests in ANA-positive children is recommended.

## Background

Approximately 20% of new patients seen at pediatric rheumatology centers are referrals for ANA positivity [1,2]. Of these, 27 to 73%, do not have an underlying connective tissue disease (CTD). General practitioners commonly send an ANA in a child with non-specific musculoskeletal complaints, such as myalgia and arthralgia. The clinical meaning of a positive ANA in a child with nonspecific musculoskeletal complaints without evidence of an autoimmune diagnosis has been a debated issue [1-3]. Several studies have followed these children overtime (3 to 5 years) and overall, they do not develop a CTD, regardless of ANA titer or pattern [1,2].

ANA has been detected in other non-CTD autoimmune diseases, such as autoimmune thyroiditis (also termed chronic lymphocytic thyroiditis or Hashimoto's thyroiditis). The frequency of ANA positivity in children with autoimmune thyroiditis has been reported in the range of 30% to 70% [4]. Possible theories for the high association of ANA with autoimmune thyroiditis are enhanced apoptosis of thyroid follicular cells, exposing nuclear antigens to elicit development of ANA, and B cell hyperactivity with production of multiple autoantibodies [5,6].

Autoimmune thyroiditis is the most common autoimmune disease in all ages, with a prevalence of 1.3 - 3.4% in children, depending on geographic location, type of study and gender of patients [7-11]. Despite the prevalence of autoimmune thyroiditis in the general population and its association with ANA, the frequency of autoimmune thyroiditis in the presence of ANA in a child without a known autoimmune disease has not been reported. Autoimmune thyroiditis, or Hashimoto's thyroiditis, can be identified by the presence of Anti-Thyroglobulin [ATG] and/or Anti-Thyroid Peroxidase [ATPO] antibodies in the serum with or without thyroid hormone abnormalities (in a euthyroid state) or clinical symptoms [7]. In our clinical experience, we have noticed an association between ANA positivity and thyroid antibody positivity in patients without a known autoimmune disease; however, this phenomenon has not been officially studied in a pediatric population. Therefore, this study was designed to addresses the prevalence of thyroid antibody positivity (Anti-Thyroglobulin [ATG] and/or Anti-Thyroid Peroxidase [ATPO] antibodies) in ANA positive children without a CTD.

## Patients and Methods

The University of Pittsburgh's Institutional Review Board approved the study prior to data retrieval. All patients younger than 18 years of age who were referred to our pediatric rheumatology center between August 2003 and March 2007 for a positive ANA with concurrent thyroid antibodies (ATG and ATPO) and thyroid function studies (TSH and FT4), and who did not fulfill criteria for a specific connective tissue disease or autoimmune disease were included. Patients with known thyroid disease were excluded.

### Laboratory evaluation

ANA was identified by HEp-2 cells, indirect immunofluorescence technique. A titer of 1:40 or higher was considered positive. ATG and ATPO were detected by ELISA, both with the cut-off level of < 60 International Units/ml for normal. TSH and FT_4 _were evaluated using age adjusted normal levels.

### Statistical analysis

Mean and standard deviation were used to describe continuous data. Chi-square or Fisher's exact tests were used to compare proportions between variables with a p-value < 0.05 considered as significant.

## Results

Two-hundred and thirty-eight patients were referred for a positive ANA and were judged not to have an underlying connective tissue disease by a pediatric rheumatologist from August 2003 to March 2007. Of these patients, 104 had thyroid antibodies (ATG and ATPO) and thyroid function studies (TSH and FT4) evaluated. The demographic, clinical and laboratory features of these 104 patients are displayed in Table 1. Ninety-three percent were Caucasian, 88% were female, and the mean age was 11.9 ± 4.0 years. Arthralgia was the most common complaint (52%). Positive thyroid antibodies were found in 31/104 (30%) of the patients. ATG was detected in 29% (30/104), ATPO in 21% (22/104) of the patients, and both antibodies were positive in 68% (21/31) of the anti-body positive patients. Ninety six percent of ATG-positive patients also produced ATPO, and 70% of ATPO-patients produced ATG.

**Table 1:** Demographic Data, Laboratory and Clinical Features

	Positive Thyroid Antibodies (n = 31)	Negative Thyroid Antibodies (n = 73)	Total (n = 104)
**Demographic features**			
Female (% within group)	30 (97)	61 (84)	91 (88)
Caucasian (%)	29 (94)	68 (93)	97 (93)
Age at first visit (yr) ± SD	12.9 ± 3.1	11.6 ± 4.2	11.9 ± 4.0
**Family history autoimmune disease**^†^			
1^st ^degree relative (%)	11 (35)	14 (19)	25 (24)
1^st ^or 2^nd ^degree relative (%)	19 (61)	35 (48)	54 (52)
**ANA pattern**			
Homogenous (%)	13 (42)	25 (34)	38 (37)
Speckled (%)	17 (55)	45 (62)	62 (60)
Nucleolar (%)	1 (3)	2 (3)	3 (3)
**Clinical manifestations**			
Arthralgia (%)	10 (32)	44 (60)	54 (52)
Rash (%)	3 (10)	9 (12)	12 (11)
Vitiligo (%)	4 (13)	2 (3)	6 (6)
Alopecia (%)	3 (10)	2 (3)	5 (5)
Fatigue (%)	2 (6)	6 (8)	8 (8)
Miscellaneous^‡ ^(%)	9 (29)	10 (14)	19 (18)

* none of the calculated p-values < 0.05

^†^Autoimmune diseases include: autoimmune thryoiditis (Hashimoto's thyroiditis), Grave's disease, rheumatoid arthritis, insulin-dependent diabetes mellitus, ulcerative colitis, Crohn's disease, systemic lupus erythematosus, psoriasis, multiple sclerosis

^‡ ^Miscellaneous manifestations include: myalgia, urticaria, thrombocytopenia, acrocyanosis

There was no statistically significant difference between the positive thyroid antibody group (n = 31) and the negative thyroid antibody group (n = 73) in regard to age at initial evaluation, ethnicity, gender or family history of autoimmune disease. Further analysis of age of onset, by using > 8 years old as an arbitrary cut-off, demonstrated that the proportion of children > 8 years old in the positive thyroid antibody group was marginally significantly higher (p = 0.06) than those in the negative thyroid antibody group (90% vs. 73% respectively); analysis not shown. Of note clinically is the higher proportion of females with positive thyroid antibodies compared to males, 33% (30/91) vs. 8% (1/13) respectively. Also demonstrated, though not significant, is a higher proportion of those in the positive thyroid antibody group with a family history of autoimmune disease compared to the negative thyroid antibody group, 61% vs. 48% respectively. The most common familial autoimmune disease in both groups was autoimmune thyroiditis, followed by rheumatoid arthritis and insulin-dependent diabetes mellitus. ANA titer and pattern did not associate with the presence of thyroid antibodies. Both groups had approximately half of the patients with a titer ≥ 320, and the pattern was speckled in 60% of all patients (Table 1).

The majority of the thyroid antibody positive patients were in euthyroid state; however, 14% had hypothyroidism requiring therapy (3 at the initial visit and 1 during follow-up). Mean duration of follow-up was 4.6 ± 9.3 months.

## Discussion

One third of the patients referred to our pediatric rheumatology center for a positive ANA without fulfilling diagnosis of CTD with concurrent evaluation of thyroid antibodies and thyroid function were found to have positive thyroid antibodies, ATG and ATPO, indicating autoimmune thyroiditis (or Hashimoto's thyroiditis). This is 7 times more frequent than the prevalence of ATG and ATPO reported in the healthy pediatric population internationally, 1.3 - 3.4% [7-11]. Therefore, our practice of screening for clinically silent autoimmune thyroiditis in ANA positive children is substantiated by the increased proportion of thyroid antibodies in these children, with potential to evolve into hypothyroidism. Older age, female gender, and family history of autoimmune disease, though not statistically significant, should raise a higher suspicion for inspection of these antibodies in the presence of a positive ANA. Although not analyzed in this study due to the retrospective design and lower prevalence of Grave's disease in the pediatric population (0.04%) [11], anti-TSH receptor antibodies should also be considered, as they are also associated with ANA positivity [5,6], but are typically identified in patients that already have clinical signs and symptoms of hyperthyroidism.

The frequency of ATG and ATPO detected in the presence of an ANA of unknown cause in adults referred to rheumatologists was reported to be 10 - 16%, which is lower than the frequency we reported here in children [12-14]. Hypothyroidism developed in the majority of these patients, 40 - 60%, within 2 years [12-14]. In children with autoimmune thyroiditis, hypothyroidism eventually developed in 20 - 50% within 5 years [7-11]. Fourteen percent of the 31 thyroid antibody-positive patients in our study developed hypothyroidism requiring therapy within our limited follow-up time (mean of 5 months). The annual incidence of a euthyroid child becoming hypothyroid in the presence of thyroid antibodies, ATG or ATPO, is 2 - 3% [10]. Proper identification of these patients is necessary for early and appropriate management.

In conclusion, we found one-third of non-CTD, ANA-positive children to have positive anti-thyroid antibodies associated with autoimmune thyroiditis, ATG and ATPO. Based on these findings and given the increased cumulative risk of euthyroid autoimmune thyroiditis in children evolving into overt thyroid disease, we recommend routine screening of anti-thyroid antibodies, ATG and ATPO, and thyroid function tests, TSH and FT4, in children with a positive ANA.

## List of Abbreviations

ANA: Anti-nuclear antibody; ATG: Anti-thyroglobulin; ATPO: Anti-thyroid peroxidase; CTD: Connective tissue disease; TSH: thyroid-stimulating hormone; FT4: free serum thyroxine.

## Competing interests

The authors declare that they have no competing interests.

## Authors' contributions

KT participated in study design, data collection, and draft of manuscript. TA conceived of the study, and performed statistical analysis. All authors read and approved the final manuscript.
